# The complete mitochondrial genome of the common dentex, *Dentex dentex* (perciformes: Sparidae)

**DOI:** 10.1080/23802359.2018.1450675

**Published:** 2018-03-22

**Authors:** Marina Ceruso, Celestina Mascolo, Giuseppe Palma, Aniello Anastasio, Tiziana Pepe, Paolo Sordino

**Affiliations:** a Department of Veterinary Medicine and Animal Production, University “Federico II”, Naples, Italy;; b Assoittica Italia, Rome, Italy;; c Biology and Evolution of Marine Organisms, Stazione Zoologica Anton Dohrn, Naples, Italy

**Keywords:** *Dentex dentex*, mitogenomics, Perciformes, Sparidae

## Abstract

The common *Dentex* (*Dentex dentex,* Linnaeus 1758) has a significant economic importance and is a highly valued table fish in the Mediterranean region. The paucity of genetic information relating to sparids, despite their growing economic value, provides the impetus for exploring the mitogenomics of this fish group. Here, we sequenced *D. dentex* complete mitochondrial genome. The sequence is comprised of 16,652 bp and consists of 13 protein-coding genes, 2 rRNA genes, 22 tRNA genes and a 2 non-coding regions (D-loop and L-origin). The overall nucleotide composition is: 27.5% of A, 28.7% of C, 26.9% of T, and 16.9% of G.

The common *Dentex* (*Dentex dentex,* Linnaeus 1758) is one of the most commercially caught fish species in the Mediterranean Sea, very appreciated in European markets. It is a littoral and benthopelagic sparid distributed in the Eastern Atlantic Ocean and in the entire Mediterranean Sea (Bauchot and Hureau 1986). Despite its significant economic importance, genetic information regarding this species is limited. We report the complete mitochondrial genome of *D. dentex* (GenBank MG727892). A specimen was caught in the Mediterranean Sea (N 41°46′58.3″, E 16°26′06.7″) and identified based on morphological features. DNA was extracted from dorsal fin tissue and is currently stored at Department of Veterinary Medicine and Animal Production, University “Federico II”, Naples, Italy. The complete mitogenome of *D. dentex* has been obtained from high-throughput sequencing on whole mitochondrial DNA with Illumina HiSeq 2500 System (Illumina, San Diego, CA, USA). The complete sequence is 16,652 bp long, containing 13 protein-coding genes, 2 ribosomal RNA genes (12S rRNA and 16S rRNA), 22 transfer RNA genes (tRNA) and two non-coding regions (D-loop and L-origin). Mitochondrial structure and gene organization are in agreement with the typical vertebrate mitogenome (Wang et al. 2008). The majority of mitochondrial genes were encoded on the heavy strand, with the NADH dehydrogenase subunit 6 (*ND6*) and eight tRNA genes [Gln, Ala, Asn, Cys, Tyr, Ser(UCN), Glu, Pro] being encoded on the light strand. Base composition is 27.5% A, 28.7% C, 26.9% T, and 16.9% G, similar to other Sparidae mitochondrial genomes (Shi et al. 2012; Dray et al. 2016). All protein-coding genes started with an ATG start codon but COI, which started with GTG. Stop codons were of 4 types, i.e. TAA (*ND1*, *ND2*, *ATP8*, *ATP6*, *COIII*, *ND4L*, *ND5*, *ND6*), AGG (*COI*), T (*COII*, *ND4*, *CYTB*) and TAG (*ND3*). The 12S and 16S rRNA genes were located between the *tRNA^Phe^* (GAA) and *tRNA^Leu^* (TAA) genes, and were separated by the *tRNA^Val^* gene as in other vertebrates (Li et al. 2016). The 22 tRNA genes vary from 66 to 74 bp in length. The 971 bp long control region is located between *tRNA^Pro^* (TGG) and *tRNA^Phe^* (GAA). The non-coding region (L-strand origin of replication) is 39 bp long and is located between *tRNA^Asn^* (GTT) and *tRNA^Cys^* (GCA).

To validate the phylogenetic position of *D. dentex*, we used MEGA6 software (Tamura et al. 2013) with the entire mtDNA sequence of the sparids *Acanthopagrus latus*, *A. schlegelii*, *D. tumifrons*, *Pagellus bogaraveo*, *Pagrus major*, *P. auriga*, *Parargyrops edita*, *Rhabdosargus sarba,* and *Sparus aurata*. The species *Lutjanus peru*, *L. rivulatus*, *Lethrinus obsoletus*, *Chaetodontoplus septentrionalis* and *Chaetodon auripes* were used as outgroup for tree rooting (Figure 1). The resultant phylogeny shows that *D. dentex* is closely related to *D. tumifrons* and *Pagrus* spp., in agreement with the work of Chiba et al. (2009). The study of the mitochondrial genome of *D. dentex* and closely related species may reveal novel barcoding regions and inform on lineage diversification patterns in sparids.

**Figure 1.**
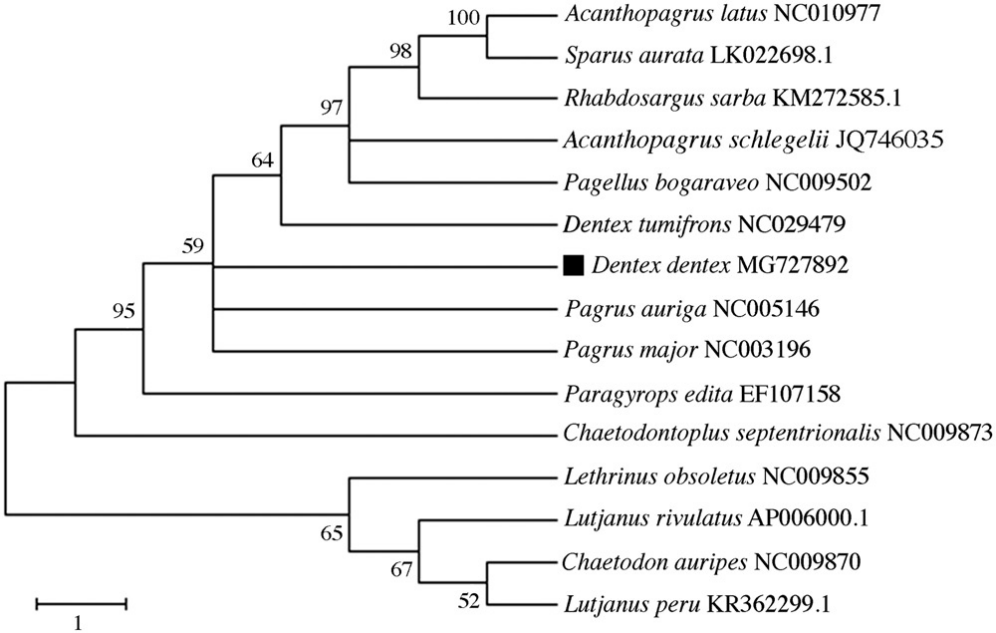
Phylogenetic analysis of *Dentex dentex* based on the entire mtDNA genome sequences of 9 sparids and 5 outgroup species by maximum likelihood method. Numbers above the nodes indicate 1000 bootstrap values. Accession numbers are shown behind species names.
